# On the Present Guideline-Focusing Points on FSCJ’s Revised Guideline for the Risk Assessment of the Effects of Food on Health for Foodborne Microorganisms and Others (Viruses, Parasites) and Its Supplemental Technical Guidance

**DOI:** 10.14252/foodsafetyfscj.D-24-00013

**Published:** 2024-12-20

**Authors:** Noriko Mizutani, Tamao Mizuno, Masako Waki

**Affiliations:** 1 Food Safety Commission Secretariat, Cabinet Office, Government of Japan, Akasaka Park Bldg 22F, 5-2-20 Akasaka, Minato-ku, Tokyo 107-6122, Japan; 2 Food Safety Commission, Cabinet Office, Government of Japan, Akasaka Park Bldg 22F, 5-2-20 Akasaka, Minato-ku, Tokyo 107-6122, Japan

**Keywords:** guideline, technical guide, microorganisms in food, food poisoning, risk assessment

## Abstract

Food Safety Commission of Japan (FSCJ) has recently revised the Guideline for the Risk Assessment of the Effects of Food on Health for Foodborne Microorganisms and Others (Viruses, Parasites) and newly issued its supplemental manual as the “Technical Guide for the Risk Assessment of the Effects of Food on Health for Foodborne Microorganisms and Others (Viruses, Parasites)”. These documents are based recent evidence and according to the Microbiological Risk Assessment Guidance for Food, Microbiological Risk Assessment Series 36 published by FAO/WHO. This short review describes the main structures and characteristics of these documents.

## Introduction

Food Safety Commission of Japan (FSCJ) has established risk assessment guidelines related to various hazards, based on the basic matters stipulated in Article 21, Paragraph 1 of the Food Safety Basic Act. The guidelines and their purpose ensure fairness and transparency of risk assessments.

In 2007, a tentative version of the Assessment Guideline for the Effect of Food on Human Health Regarding Foodborne Microorganisms, Viruses, and Parasites^1^^)^ was issued. Subsequently, in June 2021, FAO/WHO published the Microbiological Risk Assessment Guidance for Food, Microbiological Risk Assessment Series 36^2^^)^. Being consistent with such an international evaluation guidance, FSCJ had been necessary to update its guidelines. In addition, knowledge that could be integrated into the guidelines has been accumulated through the experience over the last two decades since the establishment of FSCJ. Considering these circumstances, FSCJ has recently revised the Guideline for the Risk Assessment of the Effects of Food on Health for Foodborne Microorganisms and Others (Viruses, Parasites)^3^^)^.

Additionally, FSCJ has been newly issued the “Technical Guide for the Risk Assessment of the Effects of Food on Health for Foodborne Microorganisms and Others (Viruses, Parasites)”^4^^)^ which is separated from the guideline. This guide is aimed to be used in practices of risk assessment by showing the methodology, models of evaluation, data required for the assessment, and implementation procedures with evaluation examples. Some of the contents in the tentative version of the Assessment Guideline are updated, and a plenty of recent information is described additionally. It was published in March 2023 along with the guideline. In this paper, we will introduce the contents and structure of both the Assessment Guideline and the Technical Guide.

## 2. Points on the Revision of the Guideline

### 2.1 Reconstruction of Items of the Guidelines (Table 1)

**Table 1.** Contents of the Guideline for the Risk Assessment of the Effects of Food on Health for Foodborne Microorganisms and Others (Viruses, Parasites)

Chapter 1	Background
Chapter 2	Purpose of this guideline
Chapter 3	Definitions
Chapter 4	Objective and basic concept of the assessment
Chapter 5	Scope
Chapter 6	Consideration for assessment
Chapter 7	Types of risk assessment
1	Qualitative risk assessments
2	Semi-quantitative risk assessments
3	Quantitative risk assessments
Chapter 8	Structure of risk assessment
1	Hazard identification (Identifying hazards)
2	Exposure assessment
3	Hazard characterization (Identifying adverse effects)
4	Risk characterization
Chapter 9	Simplified structure of risk assessment
Chapter 10	Use of the documents
1	Ensuring accuracy and reliability of data
2	Ensuring data transparency
3	Policy for lack of data
Chapter 11	Review of assessment
Chapter 12	Review of the guideline
References	

### 2.2 Reorganization of the Purpose and Basic Approach of Assessment:

FSCJ shall independently conduct risk assessments in an objective, neutral and fair manner based on scientific knowledge, while fully coordinating and sharing information with risk management organizations. On this guideline, FSCJ’s risk assessment for food is characterized as the “provider of risk information to risk management agencies supporting them to select risk management measures to reduce the risks.” In other words, it provides the risk managers with the “best estimate” of the risk.

### 2.3 Clarification of Target Hazards to Be Evaluated:

Microorganisms (bacteria, viruses, protozoa) and parasites other than protozoa transmitted by food were described as “microorganisms, etc.”, and the hazards to be assessment targets were “microorganisms, etc.”, toxins and metabolites produced by “microorganisms, etc.”. Food was defined as all food and beverages, including drinking water. The subjects of the assessment are human populations with hazards and adverse health effects, and the assessment will be conducted after identifying combinations of hazards and food that may contain hazards.

### 2.4 Clarification of the Characteristics of Risk Assessment of “microorganisms, etc.”:

The basic stance of the risk assessment of “microorganisms, etc.” is to reflect actual scenarios that may occur in the real world. Social and environmental factors characterizing microorganisms different from chemical substances will be considered, such as the susceptibility of the targeted population to hazards.

(1) Specific characteristics of “microorganisms, etc.”: pathogenicity, differences in strains, susceptibility to infection and transmission in humans, and dynamics through the food chain.

(2) Social and environmental factors: eating and behavioral habits of the target population; sanitary conditions of food-handling that affect the potential of intersection pollution risk.

(3) Susceptibility to hazards: differences in tolerability to the hazard in the target populations; metabolism in the human body of hazardous microorganisms and/or toxin and various interactions after ingestion between the host(human) and target microorganisms, etc., and other hazards.

## 3. Types and Components of Risk Assessment

The type of risk assessment for estimating the frequency and severity of adverse effects by the target “microorganisms, etc.” can be selected from the followings: 1. Qualitative risk assessment, 2. Semi-quantitative risk assessment, 3. Quantitative risk assessment, or the combination of any of them. The type of risk assessment will be selected depending on the purpose of the assessment as well as the quality and quantity of the data.

The risk assessment takes the approach consisted of four components based on Codex’s PRINCIPLES AND GUIDELINES FOR THE CONDUCT OF MICROBIOLOGICAL RISK ASSESSMENT (Codex: CXG 30-1999)^5^^)^: 1. Hazard identification (including information regarding the relationship and interactions of hazard, foods and the host population, as well as the causality to human diseases), 2. Exposure assessment (evaluation for the probability of human exposure to hazards via food consumption from the available information, often applying predictive microbiology models based on mathematical models), 3. Hazard characterization (identifying adverse effects), 4. Risk characterization. The goal of Risk characterization includes answering questions such as, for example, “what are the impact of the risk management measures on the risk?”

In cases of urgent needs or time constraints, however, the results of risk assessment can be presented with simplified contents of the four components depending on the circumstances.

## 4. Points Regarding Data Collection for the Assessment

The data/information for the assessment should be based on the characteristics of the target hazards, food, and host population. The important points regarding data collecting are summarized as follows.

1) Ensuring the accuracy and reliability of data: Use highly representative data

2) Ensuring data transparency: Identify sources of information

3) Policies for lack of data: Describe the data gaps between required and available for the assessment.

## 5. Technical Guide for the Risk Assessment of the Effects of Food on Health for Foodborne Microorganisms and Others (Viruses, Parasites)

This manual is issued to show the actual procedure for assessing the risk of microorganisms and others transmitted by food; and to help stakeholders to conduct rational and objective assessments. Specific methodologies and models, points of approach, and examples of the previous evaluations are presented. The structure of contents is shown in **Table 2****.**

**Table 2.** Contents of the Technical Guide for the Risk Assessment of the Effects of Food on Health for Foodborne Microorganisms and Others (Viruses, Parasites)

Chapter 1	Background
Chapter 2	Position and characteristics of risk assessment of microbiological risk analysis
Chapter 3	The Procedure of risk assessment
1	Components of risk assessment and procedures
(1)	Relationship between components of risk assessment and procedures
(2)	Risk assessment approach
(3)	Individual components of risk assessment
i	Hazard identification
ii	Exposure assessment
iii	Hazard characterization
iv	Risk characterization
2	The type of risk assessment
(1)	The type of risk assessment
(2)	Choosing the type of risk assessment
3	Statement of risk assessment results in the assessment reports
4	Data and data sources required for risk assessment
(1)	Data required for risk assessment
(2)	Sources of data collection
5	Review of the Technical Guide
Chapter 4	Details of the methods and information for risk assessment
1	Predictive microbiology
(1)	Characteristics of microbial ecology of foods
(2)	Overview of Predictive Microbiology
(3)	Basic Concepts of Growth/Death Model
(4)	Development of forecasting software
(5)	Probabilistic predictive analysis and its application
2	Dose-response
(1)	Summary
(2)	Requirement of probability theory
(3)	The developmental process of the infectious disease for dose-response
(4)	Single-hit hypothesis
(5)	Key Events Dose-Response framework
3	Sensitivity analysis
(1)	Tornado Graph
(2)	Spider Graph
(3)	Sensitivity analysis in qualitative risk assessment
(4)	Sensitivity analysis in quantitative risk assessment
4	Uncertainty and variability
(1)	Uncertainty
(2)	Variability
(3)	Variability and uncertainty statement in assessment reports
5	DALY and QALY
(1)	DALY
(2)	QALY
Chapter 5	Examples of the risk assessment
(1)	Overview of representative examples
i	Examples of semi-quantitative risk assessment
ii	Examples of Quantitative risk assessment
iii	Examples of quantitative risk assessment using new numerical indicators for risk management
iv	Example of quantitative risk assessment; a self-tasking risk assessment report by Food Safety Commission of Japan
(2)	List of evaluation examples

The main features of descriptions in this manual are listed below

**Fig. 1.**
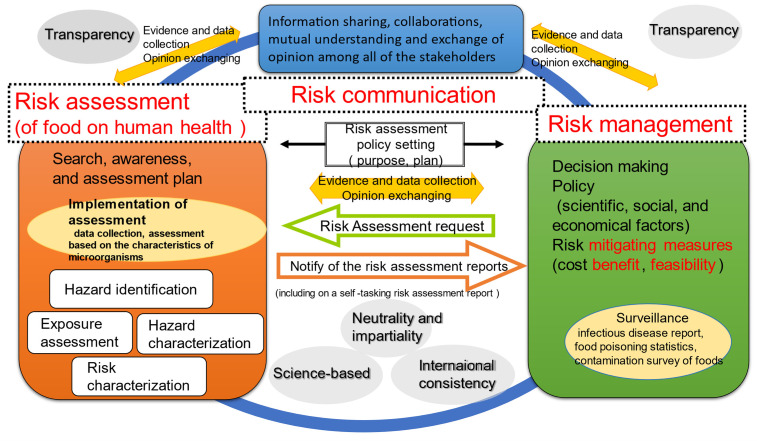
Conceptual diagram of risk analysis framework.

**Fig. 2.**
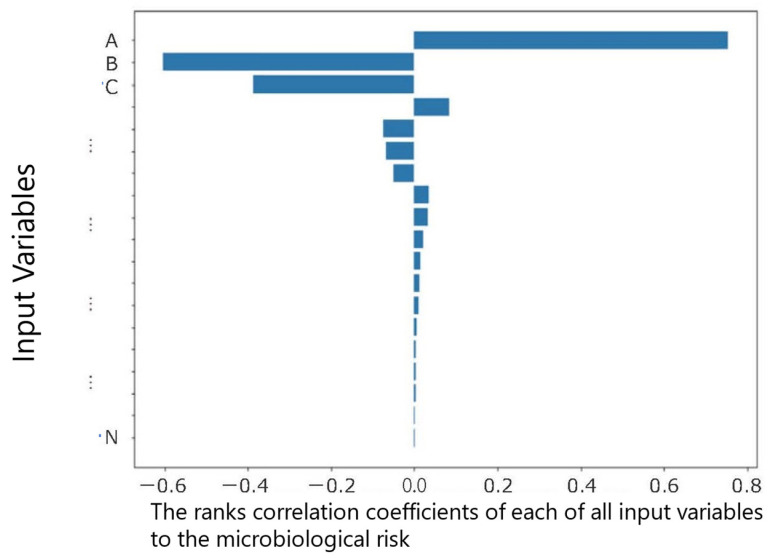
Conceptual example of sensitive analysis by Tornado Graph. The horizontal axis shows the ranks correlation coefficients of each of all input variables (A, B, C . . .) to the microbiological risk. This figure is kindly provided by Dr. Shigenobu Koseki, Professor, Research Faculty of Agriculture, Hokkaido University.

1) Containing plenty of figures and tables.

2) In Chapter 2, the position and characteristics of the assessment of microorganisms and others in food are outlined in Fig. 1***.*** It is emphasized that reducing uncertainty, improving accuracy, and promoting transparency through the cycles of PDCA/PDSA (Plan →Do→ Check→ Act/Plan→ Do→ Study→ Act.) are of importance.

3) In Chapter 3, the following examples are presented as valid approaches of the evaluation procedure: 1. baseline risk (existing level of risk) estimation, 2. comparison of risk mitigation measures, and 3. research studies/models. The choice of approach depends on the purpose of the risk assessment; adequate scenarios should be prepared. Any types of risk assessment such as qualitative, semi-quantitative, quantitative, or a combination of any of them, described in detail in this chapter, can be selected to provide risk-minimizing solutions to risk management. The document form of the assessment results is exemplified; the probability of developing the disease in a lifetime, the probability of developing the disease in one year, and the probability of developing the disease per unit of meals. Relevant data for the assessment come preferably from published literature. Information from a variety of data sources including the followings is also available; 1) statistical data of food poisoning prevalence based on the Food Sanitation Act and annual reports of the government such as detailed case reports on food poisoning incidents, 2) data of National Epidemiological Surveillance of Infectious Diseases based on the Act on the Prevention of Infectious Diseases, 3) national and international information relevant to food safety. Characteristics and sources of data required for each element of the risk assessment (hazard identification, exposure assessment, hazard characterization, and risk characterization) are summarized in a table.

4) In Chapter 4, the methods and information for the evaluation are described in detail with each example. Main subheadings are as follows; 1) Predictive microbiology (with a list of predictive microbiology tools in other countries), (2) Dose-Response, (3) Sensitivity analysis, (including Tornado graph (Fig. 2) and Spider graph), (4) Uncertainty and variability, (5) Disability-adjusted life years (DALY) and quality-adjusted life years (QALY).

5) In Chapter 5, oversea cases are shown as examples for semi-quantitative and quantitative risk assessment. In addition, FSCJ’s own experience of quantitative risk assessment in “Enterohemorrhagic Escherichia coli and Salmonella in raw meat (beef) 2011/8” and “Campylobacter jejuni/coli in chicken 2009/6” are demonstrated.

## 6. Conclusion

FSCJ has revised the “Guideline for the Risk Assessment of the Effects of Food on Health for Foodborne Microorganisms and Others (Viruses, Parasites)” and has newly issued the “Technical Guide for the Risk Assessment of the Effects of Food on Health for Foodborne Microorganisms and Others (Viruses, Parasites)” as a practical procedure manual. These documents will contribute risk assessors to implement risk assessment and prepare assessment report more accurately and rapidly. Through these documents, all of the stakeholders may deepen their awareness and understanding of this field. They will also promote to train next generation of experts in risk assessment for foodborne microorganisms.

## 7. Disclaimer Notice

The views and opinions expressed in the paper are those of the authors and should not be attributed to FSCJ.
